# Ketosis Suppression and Ageing (KetoSAge) Part 2: The Effect of Suppressing Ketosis on Biomarkers Associated with Ageing, HOMA-IR, Leptin, Osteocalcin, and GLP-1, in Healthy Females

**DOI:** 10.3390/biomedicines12071553

**Published:** 2024-07-12

**Authors:** Isabella D. Cooper, Yvoni Kyriakidou, Lucy Petagine, Kurtis Edwards, Adrian Soto-Mota, Kenneth Brookler, Bradley T. Elliott

**Affiliations:** 1 Ageing Biology and Age-Related Diseases, School of Life Sciences, University of Westminster, 115 New Cavendish Street, London W1W 6UW, UK; y.kyriakidou@westminster.ac.uk (Y.K.); l.petagine@westminster.ac.uk (L.P.); b.elliott@westminster.ac.uk (B.T.E.); 2 Cancer Biomarkers and Mechanisms Group, School of Life Sciences, University of Westminster, 115 New Cavendish Street, London W1W 6UW, UK; k.edwards3@westminster.ac.uk; 3 Metabolic Diseases Research Unit, National Institute of Medical Sciences and Nutrition Salvador Zubiran, Mexico City 14080, Mexico; adrian.sotom@incmnsz.mx; 4 School of Medicine, Tecnologico de Monterrey, Mexico City 14380, Mexico; 5 Retired former Research Collaborator, Aerospace Medicine and Vestibular Research Laboratory, Mayo Clinic, Scottsdale, AZ 85259, USA; kennethhbrookler@mac.com

**Keywords:** ageing, cortisol, GLP-1, HOMA-IR, insulin resistance, hyperinsulinaemia, ketosis, leptin, metabolic syndrome, osteocalcin

## Abstract

Metabolic dysfunctions are among the best documented hallmarks of ageing. Cardiovascular disease, Alzheimer’s disease, cancer, type 2 diabetes mellitus, metabolic-dysfunction-associated steatosis liver disease, and fragility fractures are diseases of hyperinsulinaemia that reduce life and healthspan. We studied the effect of suppressing ketosis in 10 lean (BMI 20.5 kg/m^2^ ± 1.4), metabolically healthy, pre-menopausal women (age 32.3 ± 8.9 years) maintaining nutritional ketosis (NK) for an average of 3.9 years (± 2.3) who underwent three 21-day phases: nutritional ketosis (NK; P1), suppressed ketosis (SuK; P2), and returned to NK (P3). Ketosis suppression significantly increased insulin, 1.83-fold (*p* = 0.0006); glucose, 1.17-fold (*p* = 0.0088); homeostasis model assessment for insulin resistance (HOMA-IR), 2.13-fold (*p* = 0.0008); leptin, 3.35-fold (*p* = 0.0010); total osteocalcin, 1.63-fold (*p* = 0.0138); and uncarboxylated osteocalcin, 1.98-fold (*p* = 0.0417) and significantly decreased beta-hydroxybutyrate, 13.50-fold (*p* = 0.0012) and glucagon-like peptide-1 (GLP-1), 2.40-fold (*p* = 0.0209). Sustained NK showed no adverse health effects and may mitigate hyperinsulinemia. All biomarkers returned to basal P1 levels after removing the intervention for SuK, indicating that metabolic flexibility was maintained with long-term euketonaemia.

## 1. Introduction

Ageing is typically understood in its chronological context, as the length of time that has passed since a person’s birth, whereas biological age (BA) is the measure of functional age, often measured in terms of physical and mental performance, as well as morbidities that decrease quality of life and youth-span [1,2]. One-sixth of Europeans are expected to be over 60 years of age by 2030, and 25% of older adults will be above 85 [3], reflecting a chronologically aged population increase. Concurrently, chronic non-communicable diseases promote an earlier decline in BA. Additionally, cardiovascular disease (CVD), Alzheimer’s disease (AD), and cancer are the leading causes of morbidity and mortality in the USA [4], with AD and other dementias being the leading causes for females, followed by CVD, cerebral vascular disease, and cancer in England and Wales [5]. This decline in BA was disturbingly evidenced in an analysis of 8,721 participants from 2009 to 2016 from the National Health and Nutrition Examination Survey (NHANES), showing that the proportion of metabolically unhealthy Americans increased from an already very high level of 80.1% to 87.8% over the span of 7 years, putting the vast majority of the population on the hyperinsulinaemia spectrum [2,6,7,8]. This increase in a younger BA onset and population proportion (living with morbidity earlier and for longer) is arguably the global pandemic of our time.

The chronic non-communicable diseases AD, CVD, cancer, type 2 diabetes mellitus (T2DM), metabolic syndrome (MetS), metabolic-dysfunction-associated steatotic liver disease (MASLD), and chronic inflammation are the consequence of lifestyle factors that stimulate chronic excess insulin demand and secretion, termed hyperinsulinaemia [2,7,8,9,10,11]. Hyperinsulinaemia is understood to be an ageing metabolo-endocrine state, and can be staged as metabolic phenotypes (MPs) [7,8]. Hyperinsulinaemia, when assessed as fasting insulin above the reference range only, likely does not capture the sub-clinical occult phase of hyperinsulinaemia [1]. We argue that hyperinsulinaemia can be detected before exceeding the population-derived broad reference range, which does not reflect an individual’s optimal level of insulin.

A symphony of biomarkers taken together are more sensitive to aid in catching hyperinsulinaemia as early as possible, especially in those with a body mass index (BMI) less than 25 kg/m^2^ [1,12,13,14], thus enabling earlier intervention, preventive care, and minimising the incorrect grouping of research participants, which may lead to increased false negatives in study results. A prospective cohort study from the NHANES, n = 12,563 with median age 45 years (20–85 years), found hyperinsulinaemia to be a greater risk marker for increased mortality compared to BMI [15]. We previously published a report on the effect of suppressing ketosis on ageing- and chronic-disease-associated biomarkers, which included fasting insulin, insulin-like growth factor 1 (IGF-1), glucose, beta-hydroxybutyrate (BHB), gamma-glutaryl transferase (GGT), plasminogen activator inhibitor-1 (PAI-1), monocyte chemotactic protein 1 (MCP-1), and more [7]. Part 2 investigates additional biomarkers strongly associated with morbidity, including homeostasis model assessment for insulin resistance (HOMA-IR), osteocalcin (OCN), leptin, and glucagon-like peptide-1 (GLP-1), which are biomarkers positively and negatively associated with chronic diseases and ageing. We aimed to investigate the effect of long-term sustained nutritional ketosis (NK), also known as euketonaemia, on these biomarkers, and if suppressing ketosis for 21 days would result in any measurable changes, allowing us to understand one lifestyle factor that may meaningfully impact these biomarkers, which could reduce the earlier onset of BA, meaning the potential to enhance youth-span as well as lifespan.

## 2. Materials and Methods

### 2.1. Ethics

Ethical approval was obtained by the College of Liberal of Arts and Sciences Research Ethics Committee, University of Westminster, United Kingdom (ETH2122-0634). All procedures were conducted in accordance with the Declaration of Helsinki and UK legislation. Written informed consent was obtained from all participants prior to their participation.

### 2.2. Participants

Ten healthy pre-menopausal habitually keto-adapted women, classified as metabolic phenotype 1 (MP1) [8], were recruited to take part in this three-phase study named KetoSAge [7]. The participants self-reported their adherence to a lifestyle that sustained NK for ≥ 6 months (mean 3.9 ± 2.3 years), ensuring sufficient time for metabolic adaptations. Ketosis adaptation was proven during a 6-month lead-in period, where the participants were required to take a once-daily capillary BHB reading between 4–6 p.m., before their evening meal, prior to the commencement of the study. This standardised evening measurement was chosen due to it being a more rigorous threshold to pass in order to be judged as sustaining NK over the majority of the 24 h day, in comparison to morning fasted measurements, thus increasing confidence in the participants maintaining NK most of the time. Readings were taken with a Keto-Mojo™ GKI multi-function meter (Keto-Mojo, Napa, CA, USA; [16]). The baseline characteristics of these participants were described in our earlier publication [7].

For the duration of the study, the participants were required to monitor their capillary glucose and ketone BHB concentrations (mmol/L) at four time points throughout the day to ascertain compliance (previously published in Table 3 [7]; herein expressed in rank by concentration in Table 1 in the results section). These time points were between 7:30–9:30 a.m., 11:30 a.m.–13:30 p.m., 15:30–17:30 p.m., and 21:30–23:30 p.m. The participants determined their capillary glucose and BHB using a Keto-Mojo^TM^ GKI multi-function meter. This equipment was selected for its reliability and good diagnostic performance [16].

The exclusion criteria for all participants included smoking, taking any medication, and evidence or history of metabolic syndrome, immunological issues, or CVD. The participants were required to complete a medical history questionnaire to confirm that they were free from all of the above diseases.

### 2.3. Study Design

**Figure 1:**
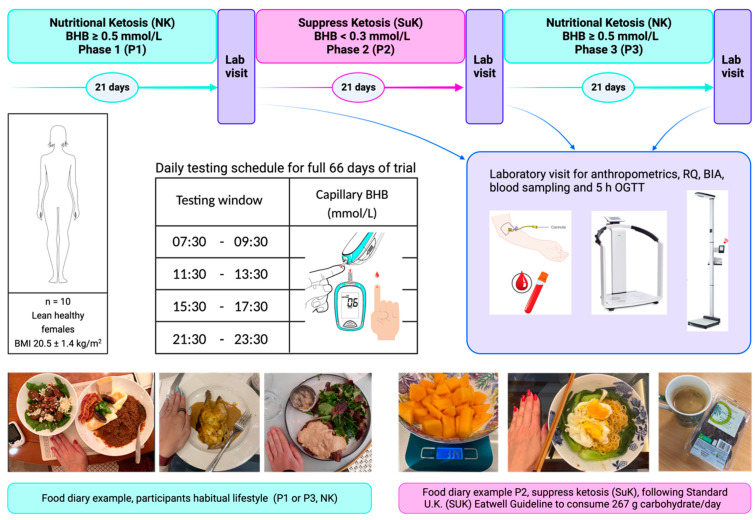
KetoSAge study design. Phase 1 and 3 covered the participants’ habitual nutritional ketosis lifestyle. Phase 2 was the interventional phase to suppress ketosis (SuK). Each phase was monitored via finger prick testing of capillary beta-hydroxybutyrate (BHB) concentration (mmol/L). Testing was conducted four times per day, prior to mealtimes, at evenly spaced intervals. At the end of each phase, participants underwent a laboratory testing day for body composition and biochemical tests. Participants were given an oral glucose tolerance test (75 g glucose in 250 mL water) described in our earlier publication [7]. Blood samples were taken at seven time points over 5 hours. Whole blood glucose and BHB were measured sequentially in real time using the Keto-Mojo^TM^ meter, and plasma insulin sensitivity assay was conducted later using ELISA. Body mass index (BMI); oral glucose tolerance test (OGGT); respiratory quotient (RQ).

The 10 KetoSAge participants took part in an open-labelled, non-randomised cross-over trial with three 21-day phases: baseline NK was defined as BHB ≥ 0.5 mmol/L (Phase 1; P1) and the suppression of ketosis (SuK) as BHB < 0.3 mmol/L, with dietary carbohydrate reintroduction following the Standard U.K. diet (SUK) Eatwell Guidelines (Phase 2; P2), which recommend consuming a predominance of calories from carbohydrates (e.g., 55% kcal from carbohydrates on a 2000 kcal diet is 275 g/day net carbohydrates). The final phase was the removal of the intervention, returning to NK (Phase 3; P3) (Figure 1). At the end of each of the 21-day study phases, on days 22, 44, and 66, the participants attended the laboratory for one day at 8 a.m. after a 12 h overnight fast to undertake anthropometric measurements and blood sampling. A detailed description of the study design was provided in our previous publication [7]. No participants withdrew from this study.

### 2.4. Anthropometric Measurements

Upon arrival at the laboratory, height (to the nearest 0.1 cm) was measured using a stadiometer (Marsden HM-250P Leicester Height Measure, Rotherham, UK), and body weight (to the nearest 0.1 kg), waist, and hip circumference measures were obtained with a non-stretch anthropometric circumference measuring tape (Seca^®^ 201, Birmingham, UK) while participants stood upright on both feet. The average value (cm) of three measurements was used for analysis. All measurements were taken from following a 12 h fast wearing standardised clothing.

### 2.5. Blood Collection and Measurement

Blood was drawn from the antecubital vein into ethylenediaminetetraacetic acid (EDTA) tubes (BD, Oxford, UK) before being centrifuged at 3,857 × *g* for 10 min at 4 °C, as described previously [7]. Plasma was then aliquoted into airtight vials and frozen at −80 °C for batch analysis later.

Samples of blood were immediately sent to SYNLAB Belgium (Alexander Fleming, 3–6220 Heppignies–Company No: 0453.111.546) for the measurement of various markers (see below). Blood was additionally drawn into serum SST^TM^ II Advance tubes with thrombin rapid clot activator and separation gel (BD, Oxford, UK), then left to stand for 30 min at room temperature. Serum tubes were centrifuged (Hettich Zentrifugen, Universal 320 R, Tuttlingen, Germany) at 3,857 × *g* for 10 min at room temperature. Serum samples were aliquoted into cryovial tubes under sterile conditions and stored at −80 °C for later analysis.

### 2.6. Blood Marker Analysis

Serum insulin was measured via Simple Plex Assay (Ella™, Bio-Techne, Minneapolis, USA). Total osteocalcin (tOCN; DuoSet, R&D Systems, Minneapolis, MN, USA), uncarboxylated osteocalcin (unOCN; BioLegend, San Diego, CA, USA), melatonin, serotonin and serum GLP-1 (Abcam, Cambridge, UK), and leptin (DuoSet, R&D Systems, Minneapolis, MN, USA) were measured by enzyme-linked immunosorbent assay (ELISA) from frozen EDTA plasma or serum samples, according to the manufacturer’s instructions. Cortisol was measured externally by SYNALB in the blood samples taken from the KetoSAge participants, as described previously [7]. Glucose concentrations were measured using a Biosen C-Line Clinic Glucose and Lactate analyser (EKF-Diagnostic, GmbH, Barleben, Germany).

### 2.7. Statistical Analysis

Data were checked for normality using the Shapiro–Wilk test. Different markers measured in the plasma between study phases for the KetoSAge participants were compared using the Friedman test with Dunn’s correction for multiple comparisons or repeated measures ANOVA with Tukey’s correction for multiple comparisons, depending on the results of the normality tests. Data are presented as mean ± SD. Data were analysed and graphed using GraphPad Prism (Version 9.1.2).

## 3. Results

### 3.1. Frequency of Rank-Ordered Capillary BHB Level

Assuming that each test was independent of the other, then the mean of a 21-day testing window had a 25% chance of being the highest. We saw in Phase 1 that 60% of the highest 21-day test window mean BHB concentrations were found in the third test of the day (pre-dinner), with the remaining 40% in the pre-lunch test (Table 1). All biomarkers assessed in this study are presented in below in Table 2.

**Table 1:** Frequency distribution of beta-hydroxybutyrate (BHB) (the mean of 21 days, BHB concentrations, per test window) expressed in rank, from 1 as the lowest to 4 as the highest concentration. Each participant took 4 tests per day, totalling 840 capillary BHB tests in 10 participants sustaining nutritional ketosis (NK) over 21 consecutive days, in two NK phases (P1 and P3), totalling 1,680 capillary BHB tests. The rank is given to the mean BHB concentration for that test window of 21 days, per phase, per participant. If a participant’s test 4 has a rank score of 1, this indicates that the mean BHB concentration for 21 days of tests at bedtime is the lowest of the mean values of all the other test windows.

**Phase 1 NK**											
Participant	1011	1021	1031	1041	1051	1061	1071	1081	1091	1101	Rank 4 frequency
Test 1 (Wake up)	2	1	2	1	2	1	1	1	1	1	0
Test 2 (Pre-lunch)	3	3	4	3	3	4	4	2	4	2	4
Test 3 (Pre-dinner)	4	4	3	4	4	2	3	4	3	4	6
Test 4 (Bedtime)	1	2	1	2	1	3	2	3	2	3	0
**Phase 3 NK**											
Participant	1011	1021	1031	1041	1051	1061	1071	1081	1091	1101	Rank 4 frequency
Test 1 (Wake up)	2	1	2	1	2	1	1	1	1	2	0
Test 2 (Pre-lunch)	4	3	4	3	2	3	4	3	3	4	4
Test 3 (Pre-dinner)	3	4	3	4	4	4	3	4	4	3	6
Test 4 (Bedtime)	1	2	1	2	3	2	2	2	2	1	0

### 3.2. Suppression of Ketosis is Associated with Increases in HOMA-IR

**Figure 2:**
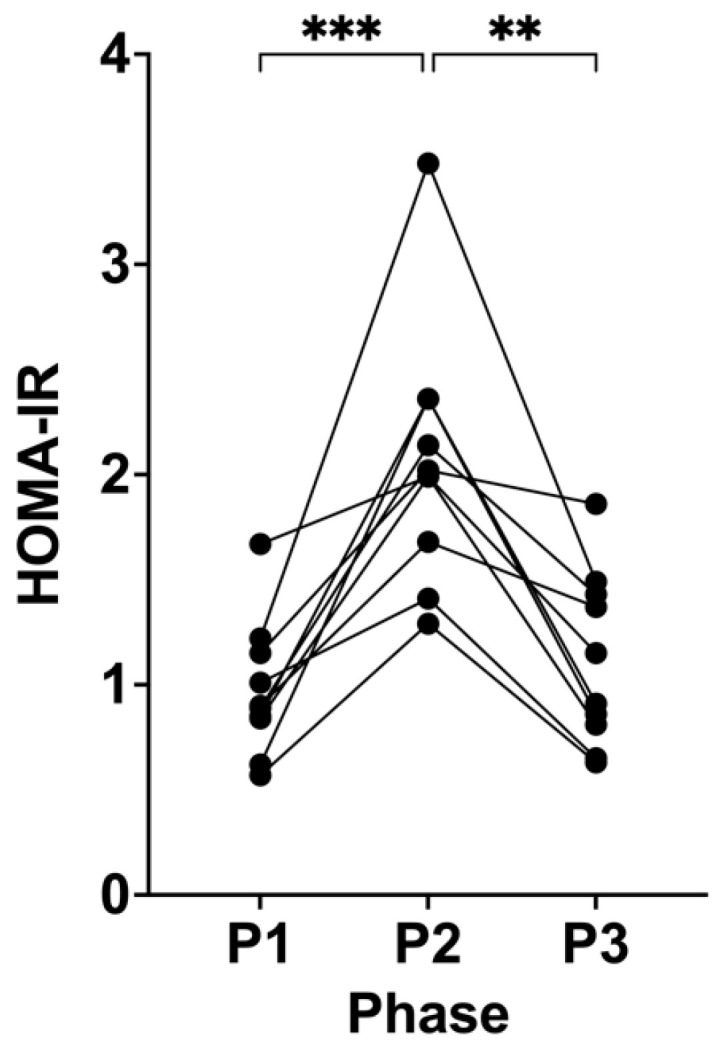
Homeostatic model assessment for insulin resistance (HOMA-IR) across all phases in KetoSAge participants. Fasting serum concentrations of insulin and plasma glucose were measured following each of the study phases: baseline nutritional ketosis (NK), P1; intervention to suppress ketosis (SuK), P2; and removal of SuK returning to NK, P3. Insulin was determined by via Simple Plex Assay (Ella™, Bio-Techne, Minneapolis, USA) and glucose was measured by Biosen C-Line Clinic Glucose and Lactate analyser. HOMA-IR adopts the following formula to index insulin resistance: fasting plasma insulin (uIU/mL) × fasting plasma glucose (mmol/L)/22.5 [1,17,18]. Homeostasis model assessment for insulin resistance (HOMA-IR). Samples were taken at 8 a.m. after a 12 h overnight fast (n = 10). Data were analysed by repeated measures one-way ANOVA. ** *p* < 0.01; and *** *p* < 0.001.

Following P2, HOMA-IR significantly increased from 0.97 (± 0.32, P1) to 2.07 (± 0.61, P2; *p* = 0.0008; Figure 2). This trend reversed following P3, where HOMA-IR significantly changed and returned to the participants’ baseline levels of 1.12 (± 0.41, P3; *p* = 0.0013) compared to P2 (Figure 2).

### 3.3. Suppressing Ketosis Increases all Forms of Osteocalcin

**Figure 3:**
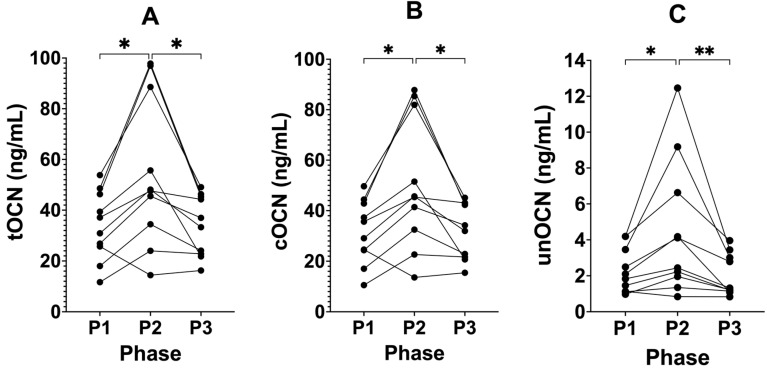
tOCN, cOCN, and unOCN across all phases in KetoSAge participants. Fasting plasma concentrations of (**A**) tOCN, (**B**) cOCN, and (**C**) unOCN were measured following each of the study phases: baseline nutritional ketosis (NK), P1; intervention to suppress ketosis (SuK), P2; and removal of SuK returning to NK, P3; total OCN (tOCN) and uncarboxylated OCN (unOCN) were determined by ELISA and carboxylated OCN (cOCN) was calculated by subtracting unOCN from tOCN. Samples were taken at 8 a.m. after a 12 h overnight fast; (n = 10); tOCN and cOCN data were analysed by repeated measures one-way ANOVA with Tukey’s correction for multiple comparisons, unOCN data were analysed using the Friedman test with Dunn’s correction for multiple comparisons. * *p* < 0.05 and ** *p* < 0.01.

tOCN significantly increased after P1 from 33.84 ng/mL (± 13.66) to 55.31 ng/mL (± 29.71, P2, *p* = 0.0138; Figure 3), and then significantly decreased following the removal of SuK (P3) to 34.02 ng/mL (± 12.05, *p* = 0.0253) compared to P2, returning to similar baseline P1 values. cOCN significantly increased after P1 from 31.54 ng/mL (± 12.59) to 50.8 ng/mL (± 26.2, P2, *p* = 0.0120), then significantly decreased following the removal of SuK in P3 to 32.0 ng/mL (± 11.1, P3, *p* = 0.0246) compared to P2, returning to similar baseline P1 values. unOCN increased after P1 from 2.29 ng/mL (± 1.25) to 4.54 ng/mL (± 3.79, P2, *p* = 0.0417), then decreased following the removal of SuK in P3 to 2.00 ng/mL (± 1.16, *p* = 0.0010) compared to P2, returning to similar baseline P1 values.

**Figure 4:**
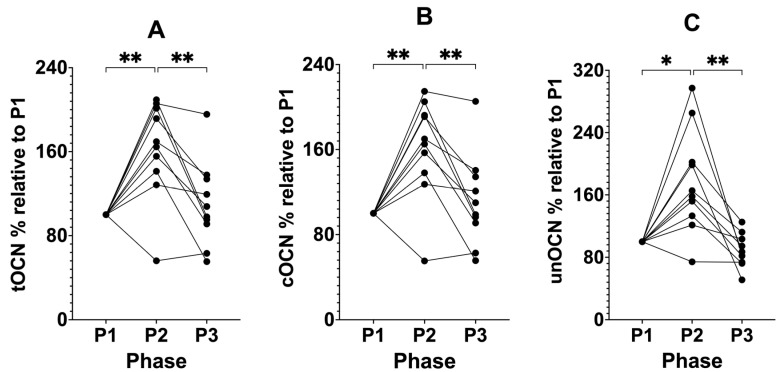
Percentage change from baseline P1 at 100% for tOCN, cOCN, and unOCN across all phases in KetoSAge participants. Fasting plasma concentrations of (**A**) tOCN, (**B**) cOCN, and (**C**) unOCN were measured following each of the study phases: baseline nutritional ketosis (NK), P1; intervention to suppress ketosis (SuK), P2; and removal of SuK returning to NK, P3; total OCN (tOCN) and uncarboxylated OCN (unOCN) were determined by ELISA, carboxylated OCN (cOCN) was calculated by subtracting unOCN from tOCN. Samples were taken at 8 a.m. after a 12 h overnight fast; (n = 10); data were analysed by repeated measures one-way ANOVA with Tukey’s correction for multiple comparisons. * *p* < 0.05 and ** *p* < 0.01.

We analysed tOCN, cOCN, and unOCN as percentages relative to each participant’s own baseline values in P1. tOCN significantly increased after P1 from 100% as the standardised baseline (± 0.00%) to 162.36% (± 46.53%, *p* = 0.0065; Figure 4) relative to P1, then decreased following the removal of SuK in P3 to 109.70% (± 40.36%, P3, *p* = 0.0094), relative to baseline and compared to P2, returning to similar baseline P1 values. cOCN significantly increased after P1 from 100% as the standardised baseline (± 0.00%) to 161.56% (± 46.73%, P2, *p* = 0.0062) relative to P1, then decreased following the removal of SuK in P3 to 111.52% (± 42.90%, P3, *p* = 0.0080), relative to baseline and compared to P2, returning to similar baseline P1 values. unOCN significantly increased after P1 from 100% as the standardised baseline (± 0.00%) to 176.67% (± 66.66%, P2, *p* = 0.0135) relative to P1, then decreased following the removal of SuK in P3 to 87.11% (± 21.43%, P3, *p* = 0.0076), relative to baseline and compared to P2, returning to similar baseline P1 values.

### 3.4. Suppression of Ketosis is Associated with Increased Leptin

**Figure 5:**
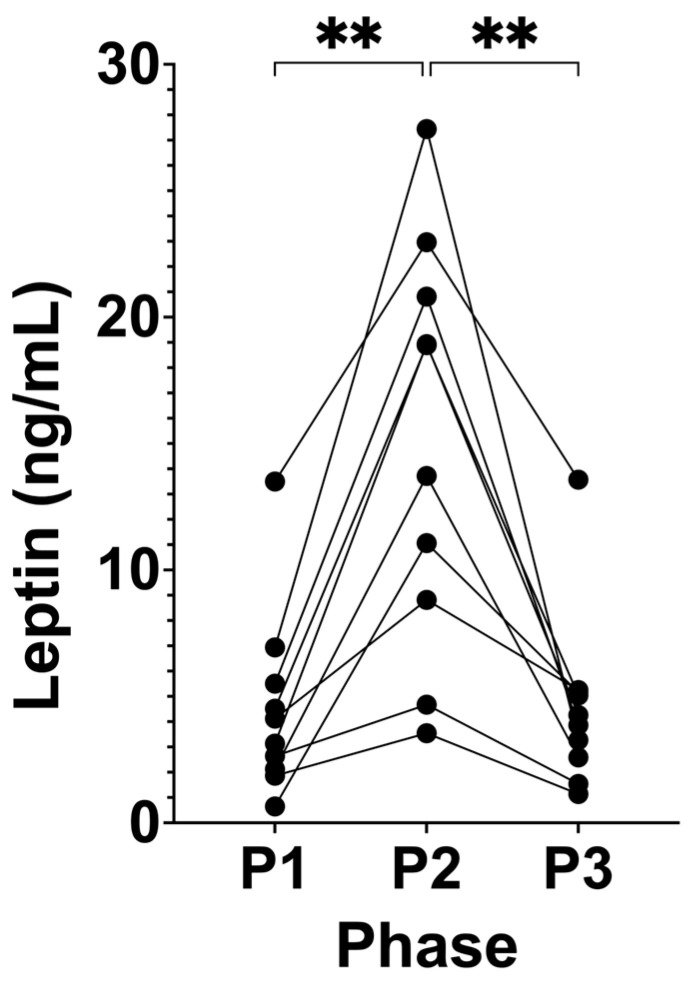
Serum leptin across all phases in KetoSAge participants. Fasting serum concentrations of leptin were measured following each of the study phases: baseline nutritional ketosis (NK), P1; intervention to suppress ketosis (SuK), P2; and removal of SuK returning to NK, P3. Leptin was determined by via ELISA (DuoSet, R&D Systems, Minneapolis, MN, USA). Samples were taken at 8 a.m. after a 12 h overnight fast; (n = 10). Data were analysed by repeated measures one-way ANOVA. ** *p* < 0.01.

Following P2, leptin significantly increased from 4.50 ng/mL (± 3.67, P1) to 15.08 ng/mL (± 8.00, P2; *p* = 0.0010; Figure 5). This trend reversed following P3, where leptin significantly decreased and returned to the participants’ baseline levels of 4.57 ng/mL (± 3.48, P3; *p* = 0.0005) compared to P2.

### 3.5. Cortisol Levels Remain at Healthy Low Levels in Long-Term NK, and Serotonin Does Not Significantly Change

**Figure 6:**
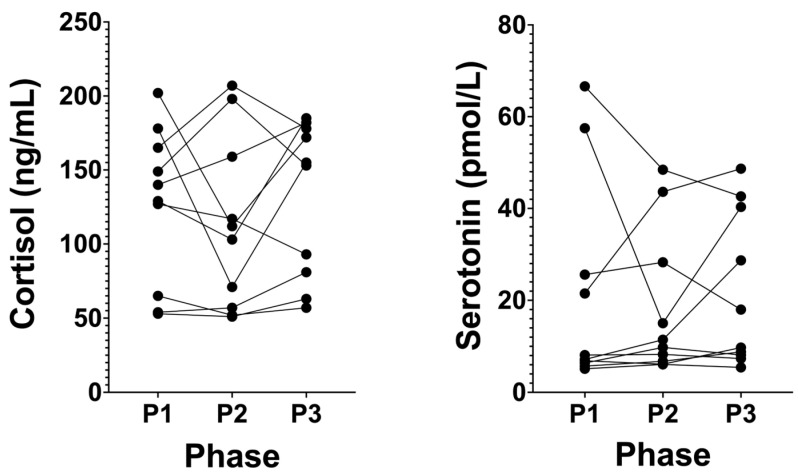
Serum cortisol and serotonin across all phases in KetoSAge participants. Fasting serum concentrations of cortisol and plasma serotonin were measured following each of the study phases: baseline nutritional ketosis (NK), P1; intervention to suppress ketosis (SuK), P2; and removal of SuK returning to NK, P3; cortisol was measured externally by SYNLAB Belgium (Alexander Fleming, 3–6220 Heppignies–Company No: 0453.111.546), serotonin was measured by ELISA (Abcam, Cambridge, UK). Samples were taken at 8 a.m. after a 12 h overnight fast; (n = 10). Data were analysed by Friedman test with Dunn’s correction for multiple comparisons.

Following P2, cortisol did not significantly change after P1, changing from 126.20 ng/mL (± 52.67, P1) to 112.70 ng/mL (± 58.46, P2; *p* ≥ 0.9999; Figure 6). Similarly, following P3, there was no significant change compared to P2 at 131.90 ng/mL (± 52.18, P3, *p* = 0.5391). Following P2, plasma serotonin did not significantly change from P1 at 21.05 pmol/L (± 22.83, *p* = 0.7907; Figure 6). With the removal of the intervention for SuK with a return to NK for 21 days at the end of P3, there was no significant change in serotonin compared to P2 at 21.77 pmol/L (± 16.80, P3, *p* ≥ 0.9999).

### 3.6. Suppression of Ketosis is Associated with Decreased GLP-1

**Figure 7:**
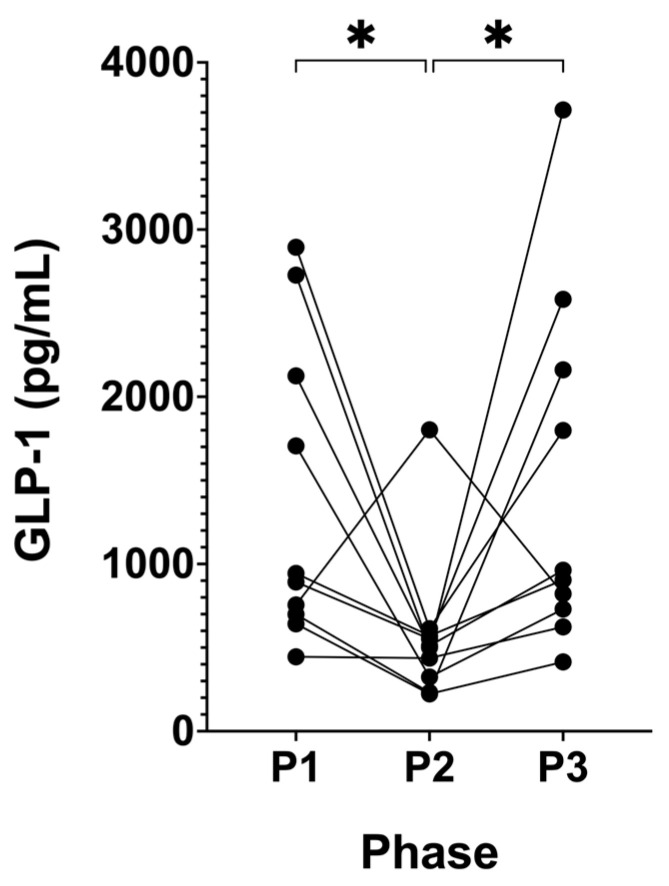
Serum concentrations of active GLP-1 across all phases in KetoSAge participants. Serum concentrations of active GLP-1 were measured following each of the study phases: baseline nutritional ketosis (NK), P1; intervention to suppress ketosis (SuK), P2; and removal of SuK returning to NK, P3. GLP-1 was determined by ELISA (Abcam, Cambridge, UK). Glucagon-like peptide-1 (GLP-1). Samples were taken at 8 a.m. after a 12 h overnight fast; (n = 10). Data were analysed by repeated measures one-way ANOVA. * *p* < 0.05.

Following P2, active GLP-1 significantly decreased from 1,064.98 pg/mL (± 500.15, P1) to 504.98 pg/mL (± 246.43, P2; *p* = 0.0209; Figure 7). This trend reversed following P3, where GLP-1 significantly increased and returned to the participants’ baseline levels of 1,032.26 pg/mL (± 453.88, P3; *p* = 0.0210) compared to P2.

## 4. Discussion

### 4.1. When to Measure Capillary BHB

The 6-month 4–6 p.m. capillary ketone testing standardised evening measurement as a criterion for eligibility for the trial was chosen due to it being a more rigorous threshold to pass in order to be judged as sustaining NK over the majority of the 24 h day, in comparison to morning fasted measurements, thus increasing the confidence in the participants maintaining NK most of the time. Most people embarking on a ketogenic lifestyle, typically via carbohydrate restriction, time-restricted feeding, or other forms of fasting-mimicking diets, will measure their capillary ketones in the morning, much the same way that morning fasting blood glucose is taken. However, morning ketone readings for hyperinsulinaemic and insulin-resistant individuals will likely produce some measurable levels (potentially up to 0.5 mmol/L) due to an overnight fast. If this was performed on individuals only daily, they could mistakenly then assume that they are successfully maintaining a metabolic state of ketosis. However, if they were to measure their capillary ketones at least 3 to 4 hours after lunch, then this test would capture their breakfast and/or lunch if they had consumed anything, allowing them to truly know if their lifestyle was supportive of maintaining euketonaemia via minimising lifestyle practices that stimulate excess insulin demand, which would inhibit (lower) ketone production.

In our cohort, our participants noted that their pre-lunch and dinner readings were often their highest levels of ketones out of the four times a day tests, with their pre-dinner (4–6 p.m.) reading showing a slightly higher frequency of being greater than their second test of the day (pre-lunch). This informed our participants of how their lifestyle was supporting them in maintaining nutritional levels of ketones (≥ 0.5 mmol/L) throughout the day [7].

### 4.2. Hypoketonaemia, HOMA-IR, and Fasting Insulin

Throughout the KetoSAge study, the participants maintained healthy fasting glucose levels in all phases, 4.36 mmol/L (± 0.53, P1), 5.12 mmol/L (± 0.59, P2), and 4.41 mmol/L (± 0.30, P3), which are considered to be extremely healthy [17]. HbA1c was not measured in this study, due to the three-phase blood sampling timing that would have resulted in prior blood sampling affecting the next blood sampling results (P2 affected by P1, and P3 affected by P2). HbA1c is used to assess cumulative glucose exposure, which glycates the haemoglobin (HbA1c) in blood. This provides evidence of average blood glucose levels over the previous two to three months, which is the average half-life of red blood cells [18]. A percentage of glycation damage occurs as a result of hyperglycaemia, which is associated with an increased risk of atherosclerosis, CVD, cerebrovascular disease, all-cause dementia (ACD), and AD. For example, in the AgeCoDe cohort study of 1,342 elderly individuals, a higher HbA1c level was found to be associated with increased ACD and AD. HbA1c ≥ 6.5% was associated with an increased risk of ACD by 2.8-fold (*p* = 0.027) and for AD (*p* = 0.047). There was an associated 5-fold increased risk of incident ACD (*p* = 0.001) and a 4.7-fold increased risk of AD (*p* = 0.004) with HbA1c levels ≥ 7% [19]. However, hyperinsulinaemia has been shown to precede hyperglycaemia by up to 24 years [20]. Therefore, there is a weakness when relying on HbA1c to detect earlier disease risk, in that there is a sub-clinical phase (occult stage), during which, a healthy HbA1c value is seen. This is due to an increased insulin concentration that reduces the plasma glucose, whilst the elevated basal insulin still has non-glucose-related signalling effects, such as increasing PI3K and MAPK signalling for growth and division [21,22,23,24] and increasing ceramide production, which increases mitochondrial reaction oxygen species generation, therefore increasing oxidative stress in the system [25,26,27].

An example of how a reduced HbA1c due to more insulin exposure can be less positive for health is found in the ACCORD group randomised controlled trial (RCT) study of 10,251 patients, with a mean age 63 years, who had an 8.1% median HbA1c. All participants were already on exogenous insulin. Half of the group was randomised to a more tailored intensive insulin therapy regimen to achieve a decrease in their HbA1c to 6%, whilst the other group was to follow their standard insulin therapy to target a value of 7.0–7.9%. The three primary outcomes measured in this RCT were non-fatal stroke, non-fatal myocardial infarction, and CVD death. This trial was stopped after 3.5 years due to ethical reasons, as the intervention group receiving the intensive insulin therapy (more insulin) in order to lower their HbA1c by a greater amount had a significantly higher mortality rate [28]. Whilst successfully lowering their HbA1c with more insulin, there was an increase in deaths, indicating that relying on a lower HbA1c may lure physicians and patients into a false sense of security whilst patients are actually exposed to greater insulin levels and, therefore, their subsequent potential harms [2,8,24,27,29,30,31].

HOMA-IR is recognised as a diagnostic index for insulin resistance, where a higher concentration of fasting insulin to maintain a low fasting glucose results in a higher HOMA-IR value [1]. Insulin resistance is generally accepted as having an HOMA-IR > 2 [32,33,34], whilst hyperinsulinaemia is considered where the fasting plasma insulin is > 28.8 uIU/mL [8,9,22,35]. Our participants’ group mean of fasting insulin levels was 9.06 uIU/mL (± 2.13) after 21 days of suppressing ketosis, with the highest individual fasting insulin measuring a value of 11.92 uIU/mL. However, HOMA-IR was able to detect a significant change, where, in both NK phases, our participants’ HOMA-IR was at the very healthy levels of 0.97 (± 0.32, P1) and 1.11 (± 0.41, P3). Conversely, suppressing ketosis managed to significantly double their mean HOMA-IR to 2.07 (± 0.61, P2), bringing it into the beginning range of insulin resistance, with one participant having an HOMA-IR of > 3 after suppressing ketosis for 21 days. All participants’ HOMA-IR values returned to their healthy baseline values within only 21 days of returning to NK by removing the intervention for SuK via the ingestion of carbohydrates. Given HOMA-IR’s strong association and even predictive value in chronic disease risk, it would be of high value to understand how lifestyle may modulate this marker index measured alongside other biomarkers strongly associated with disease risk, such as GGT, IGF-1, MCP-1, and ageing, to provide further confidence and resolution in diagnostics and the more accurate phenotyping/categorisation of patients in studies [7].

In a cross-sectional analysis study of 12,266 participants from the NHANES database, a positive correlation was found between participants’ HOMA-IR levels and their BA in US adults [1]. A prospective cohort study of 3,741 asymptomatic employees of Santander Bank in Madrid, aged between 40–55 years upon recruitment, with no known CVD, investigated the relationship between early insulin resistance, as measured using the HOMA-IR index in normoglycaemic individuals assumed to be at a low risk of atherosclerosis, in addition to the effect of cardiovascular risk factors in individuals with normal HbA1c. The presence and progression of sub-clinical atherosclerosis (SA) were assessed using non-invasive vascular imaging modalities, multiterritorial vascular ultrasound, and coronary arterial calcification (CAC) scans [36]. The study found that HOMA-IR had a direct association with the multiterritorial extent of SA and CACs (*p* < 0.001). In summary, 85.1% of the reference group (HOMA-IR < 2) were free of coronary artery calcium (14% had a CACS of > 0). This proportion of participants with CAC increased as insulin resistance increased, with a HOMA-IR between 2 to 3, the proportion increased to 25.6%, and with a HOMA-IR > 3, the proportion increased further to 39.3%. A bibliometric study of 1,500 publications found HOMA-IR insulin resistance to be strongly associated with cerebral small vessel disease, ischemic stroke incidence, post-stroke depression, and early neurological deterioration in patients who suffered stroke [29].

The 21 days of suppressing ketosis resulted in hypoketonaemia due to insulin’s suppressive control of ketogenesis [28]. Whereas P1 and P3 were typified by euketonaemia, along with healthy low insulin and HOMA-IR values. As our cohort was exclusively female, it is imperative to recognise the links between hyperinsulinaemia, metabolic health, and breast cancer [4,30]. In a retrospective case–control study, 80 non-diabetic patients with pre-menopausal and post-menopausal breast tumours were compared to 60 women with normal mammograms as a control. Hyperinsulinaemia and insulin resistance HOMA-IR have been found to be associated with an increased risk of breast cancer in non-diabetic (normoglycaemic) women. Whilst hyperinsulinaemia determined by fasting insulin levels of > 28 uIU/mL may proceed hyperglycaemia for up to 24 years [2,7,8,9,31], early sub-clinical (occult) hyperinsulinaemia may be considered when there is chronic hypoketonaemia measured between 4–6 p.m. for at least three consecutive days (a longer duration would increase confidence). Measurement at this time of day would reduce the false-positive acceptance of NK compliance from a test result resulting from waking up with ketosis levels (> 0.3 mmol/L) due to an overnight fast [7,32].

In our KetoSAge trial, the chronic suppression of ketosis (P2) intervention resulted in significantly increased fasting insulin levels, which would still be considered as healthy values, yet significantly increased HOMA-IR values into the insulin resistant range. Our participant data show that long-term NK maintains and reduces HOMA-IR to very healthy levels, adding to existing evidence that sustaining a lifestyle that promotes ketosis (minimising insulin demand and secretion) is an effective modality in preventing insulin resistance and its associated diseases, including cancer, CVD, dementia, T2DM, and earlier biological ageing [1,7,9,33,34,35]. It was very reassuring to see that our participants’ HOMA-IR levels returned to their baseline mean value of 1.11 (± 0.41, P3) after the removal of carbohydrates, returning to sustained NK, indicating that restricting carbohydrates is a viable and practical method to reduce the risk of insulin-resistance-related morbidities known to affect healthspan and lifespan. Additionally, our data indicate that the effect of 21 days of suppressing ketosis on HOMA-IR, can be reversed by a subsequent period of sustained NK, in individuals who habitually maintain a ketogenic lifestyle.

### 4.3. Osteocalcin

Human OCN has a molecular weight of 5 kDa, is 49 amino acids long, and is the tenth most abundant protein in the body [37]. It has been shown to have an endocrine function in glucose homeostasis, insulin sensitivity, neurogenesis, cognitive health, and mitochondrial biogenesis [38,39,40,41,42,43]. The majority of OCN is synthesised by osteoblasts and osteocytes in the bone. Its carboxylated form (cOCN) is both deposited into the bone and released into the system by osteoblasts and osteocytes. unOCN is synthesised and released into the circulation by osteoblasts and osteocytes, as well as resorbed from the bone by osteoclasts [39]. cOCN is required for the correct alignment of hydroxyapatite (HA), which confers bone its torsion and tensile ductility, giving it resistance to fragility fractures, which is not captured via bone mineral density (BMD) assessment [39,44].

In the KetoSAge trial, all forms of osteocalcin (tOCN, cOCN, and unOCN) significantly increased after 21 days of suppressing ketosis and then decreased back to baseline with the return to ketosis. Interestingly, OCN is synthesised and secreted from adipocytes only during adipogenesis [45]. Weight gain as fat mass occurred during the 21-day intervention phase (P2) to suppress ketosis via the re-introduction of dietary carbohydrates following the SUK Eatwell Guideline recommendations (data previously published in [7]). This may explain the increases in all forms of OCN.

tOCN, cOCN, and unOCN increased from P1 to P2 in all but one participant (ID: 1091). From P2 to P3, tOCN and cOCN decreased in all but one participant, which was the same individual (ID: 1091). Upon further investigation, participant ID: 1091 regularly reported consuming beef bone broth during P1 and P3. Since OCN is the second most abundant protein in bone, it is plausible that this broth may have served as an exogenous source of OCN, thus explaining the increase [38]. Removal of their data in unOCN analysis results in a significant decrease from P2 at 4,691.87 pg/mL (± 3,751.66 pg/mL) to P3 at 1,873.10 pg/mL (± 1,173.00 pg/mL; *p* = 0.0489). Further investigation into the exposure of exogenous OCN from foodstuffs is, therefore, warranted.

An increased HOMA-IR increases coronary atherosclerosis risk; furthermore, insulin resistance has a sub-clinical occult phase potentially detectable via repeated consecutive days blood ketosis and glucose testing (between 4–6 p.m.), where a capillary BHB value of < 0.3 would indicate hypoketonaemia due to occult hyperinsulinaemia. Endothelial progenitor cells (EPCs), which aid in the repair of the vasculature, may also come with vascular calcification, as they have been shown to express OCN genes in insulin-resistant chronic disease conditions such as atherosclerosis [36]. EPCs from 72 patients with coronary atherosclerosis undergoing invasive coronary assessment have been shown to express OCN genes, indicating epigenetic osteogenic transformation [46]. Other studies of patients with hyperinsulinaemia conditions, such as in CVD and chronic kidney disease, have detected vascular smooth muscle cell osteogenic differentiation and aortic valve tissue having an increased expression of sclerostin [39,47,48,49,50], corroborating the relationship between chronic increased insulin exposure altering gene expression in the osteogenic profile.

In a study of 2,493 individuals with MetS, plasma OCN levels were found to be inversely correlated with HOMA-IR, fasting insulin and glucose, leptin, and BMI (*p* < 0.0010 for each biomarker) [51]. Interestingly, OCN has been found to be lower in obese people [52,53] and those with T2DM, such as seen in a correlation analysis of 204 patients with T2DM, where an inverse relationship between OCN and HOMA-IR was found [52,54]. With this information, it would be easy to then make the assumption that a higher level of OCN would, therefore, be better. However, our KetoSAge trial showed an opposite pattern, where P1 and P3 had the healthiest low HOMA-IR values and significantly lower OCN (all forms) relative to P2, indicating OCN was positively associated with HOMA-IR in this cohort. Furthermore, as BMI and fat mass increased in P2 [7], so too did all forms of OCN in the present study. In corroboration with Ferron’s work [55], OCN increased in P2, where insulin also increased, indicating a potential effect of OCN increasing insulin secretion. However, our trial did not corroborate an increase in sensitising glucose uptake, given that P2 came with a significant increase in fasting blood glucose, although the mean value was still in a healthy range. It is possible that OCN was rescuing the situation, meaning that, if OCN did not increase, perhaps there would have been a greater increase in glycaemia and a trend towards IR. The OCN patterns in MP1 individuals may behave much like free triiodothyronine (T3), where T3 demand increases as mitochondrial reactive oxygen species increase, causing oxidative damage to mitochondrial (mt) oxidative phosphorylation (OXPHOS) proteins and resulting in an increased demand of T3 to upregulate the synthesis of mtOXPHOS electron transport chain proteins. Furthermore, like the pattern seen in our prior work [7,32], we saw a lower T3 when in long-term ketosis and an increase in T3 after 21 days of suppressing ketosis, likely indicating an increased T3 sensitivity and decreased demand, which may be a similar case to OCN. If we assume that, in a healthy setting, OCN would be synthesised and deposited into the bone and that only a low dose of OCN is steadily released into the circulation, therefore, bone OCN deposition would confer a healthier bone tensile strength, and there may be a potential greater sensitivity to the OCN in the circulation [7,32].

OCN has been shown to stimulate GLP-1 synthesis [56], a pattern that was not corroborated in our trial. However, there may be a difference between endogenous versus oral exposure to OCN. It is interesting that, as OCN significantly increased in P2, GLP-1 conversely decreased, which is an opposite trend to other experiments showing OCN activating GLP-1 synthesis [56,57]. Research into human physiology often investigates pathology and tries to reverse engineer relationships, or very often uses a control group labelled as the “healthy” group, where, in actual fact, they are the “common” group and not necessarily a group reflecting human evolutionary phenotypic living, which is likely to be in a state of ketosis for the majority of each 24 h day [2,8,58]. This would result in a metabolic signature that is different to non-pathology-presenting long-term suppressed ketosis metabolic phenotype 2 (MP2) people [8]. A low OCN concentration in healthy long-term ketosis should not be confused with a low OCN level as seen in obesity, T2DM, CVD, and AD, where these cohorts are also seen to have higher rates of fragility fractures with a normal to high bone mineral density, which is hyperinsulinaemia osteofragilitas and not osteoporosis (Cooper, Brookler and Crofts, 2021 [39]). This indicates poor bone health, with hyper mineralisation as a form of osteocyte fossilisation, rendering them unable to thrive nor survive, nor produce the necessary OCN, not only for the alignment of HA to confer bone tensile strength, but to also to be released into the circulation to play its part in healthy metabolic homeodynamic regulation [39]. In this context, a low plasma OCN concentration is indicative of pathology. It is, therefore, clear that clinicians and researchers need to be aware of understanding biomarkers within the metabolic phenotype profile of each individual, so as not to misdiagnose/categorise according to a single marker out of the patient’s metabolic context.

### 4.4. Leptin Increases with Chronic Suppression of Ketosis a Risk of Hyperleptinaemia

Leptin is an adipokine, synthesised and secreted by white adipocytes, which has a 24 h circadian rhythm [59] and may be a more sensitive marker for hyperinsulinaemia than BMI. In a study of 119 normal-weight 18- to 24-year-old participants with a BMI of < 25, it was found that fasting leptin levels were significantly associated with fasting insulin (β ± SE = 0.30 ± 0.06, *p* < 0.001) and HOMA-IR (β ± SE = 0.41 ± 0.20, *p* < 0.001) [14]. These relationships were independent of age, gender, and total body fat. This indicates that, although leptin increases with obesity, it may be, that which causes obesity or excess body fat, which can exist in normal-BMI individuals, often termed TOFI (thin on the outside, fat on the inside) [60,61], also causes hyperleptinaemia and hyperinsulinaemia. These share a common root cause, and, therefore, explain why we see leptin and insulin rising where obesity may not, or may rise later on, meaning that obesity was not the first mover in causing the rises in these biomarkers associated with chronic metabolic diseases. Chronic excess insulin is the likely root cause, since insulin is so intrinsically linked to the regulation of leptin, and that excess may not be captured by a fasting insulin nor an oral glucose tolerance test (OGTT). Insulin tightly regulates ketogenesis, therefore, many consecutive days of BHB testing between 4–6 p.m. acts as a proxy to understanding individual hyperinsulinaemia, if we accept hyperinsulinaemia to mean “more than is ideal for optimal health” for an individual [28,62]. Our KetoSAge participants’ leptin levels were healthily low in their natural habitual ketosis states, at levels classified/categorised as the healthiest in terms of CVD risk [63]. With the 21-day suppression of ketosis, which resulted in a significant increase in fasting insulin, there was a significant increase in fasting leptin too, to levels that re-classify into less healthy ranges associated with poorer health outcome [63]. Returning to ketosis showed a complete return to baseline levels, indicating how responsive leptin is to dietary carbohydrate-stimulated insulin secretion in long-term habitually keto-adapted females.

The KetoSAge trial participants’ weight and fat mass did increase significantly after 21 days of SuK, however, their BMI stayed well within the healthy range. This corroborates the understanding that, although BMI is a good tool for investigating disease risk, there are subsets of people with sub-clinical (occult) conditions that are being pooled into research, which decreases accurate reflections of physiology and pathophysiology in trial findings and data analysis. A BMI of < 25 kg/m^2^ is considered as healthy, however, there are subsets of people with a normal BMI who have metabolic diseases and even some with a BMI of > 25 kg/m^2^ that may have occult hyperinsulinaemia for many years when their BMI was < 25 kg/m^2^ [60,61,64,65,66]. Boden et al. showed, using a 72 h euglycaemia-hyperinsulinaemia and hyperglycaemic clamp, that prolonged hyperinsulinaemia and not hyperglycaemia increased serum leptin in 28 healthy normal-weight males. Furthermore, high levels of free fatty acids also did not affect leptin release [59]. Whilst this trial intervention for SuK for 21 days resulted in these significant changes, it is thought-provoking to consider if it had lasted months, if not years.

Higher levels of leptin have been shown to be associated with increased frailty in older adults. Fragility fracture rate increases with hyperinsulinaemia [39], which is also associated with ageing, given that the “chronic” in chronic-elevated insulin indicates a long duration of time exposure, which chronological age reflects. In an investigation between leptin levels and incident frailty in 1,573 individuals from the Seniors-ENRICA cohort, aged ≥ 60 years and without T2DM [67], those with leptin levels in the highest tertile had a significantly increased risk of frailty (odds ratio [OR]: 2.12; 95% confidence interval [CI]: 1.47–3.06; *p*-trend < 0.001). In a multivariate linear regression analysis, leptin levels were shown to be positively associated with insulin resistance in 398 middle-aged and elderly Taiwanese individuals (β = 0.226, *p* < 0.01) [68]. Leptin has also been shown to be positively associated with atherosclerosis assessed by CAC in a cross-sectional study on 200 participants, aged between 35 and 75 years with T2DM (which is hyperinsulinaemia with hyperglycaemia, with insulin resistance) [63]. Given that the HOMA-IR and progression of early SA-CNIC-Santander study (NCT01410318) showed a significantly increased risk of SA with an increase in HOMA-IR, the KetoSAge trial provides us with valuable information on increasing our knowledge about how to maintain or modulate these biomarkers associated with one of the global leading causes of chronic disease mortality, CVD [36]. Our KetoSAge trial showed lower leptin levels during ketosis, coupled with very low HOMA-IR (no insulin resistance), and the suppression of ketosis increased leptin in lockstep with increasing insulin resistance. The removal of dietary farinaceous carbohydrates to no longer stimulate an increased insulin demand repeatedly showed how responsive these chronic disease biomarkers are to ketosis, the metabolic state that, throughout history, humans likely spent most of their time in.

### 4.5. Cortisol and Serotonin

The steroid glucocorticoid hormone cortisol is mainly synthesised in the adrenal cortex by the zona fasciculata and, to a much lesser degree, by the thymus, brain, intestine, and skin [69]. Further, cortisol is involved in the regulation of gluconeogenesis and is often considered to be the stress response or related hormone [70]. In 919 participants aged 60–75 years from the Edinburgh Type 2 Diabetes prospective study, elevated fasting plasma cortisol was positively associated with increased ischaemic heart disease [71]. The univariate analysis found an associated increased odds of ischaemic heart disease with cortisol concentrations of > 800 nmol/L (290 ng/mL). The standard reference range for cortisol in plasma is 33–246 ng/mL [72]. Our KetoSAge trial mean fasting serum cortisol levels were within healthy ranges in all phases of the trial, with ketosis phases’ mean concentrations of 126.20 ng/mL (± 52.67, P1) (348.13 nmol/L) and 131.90 ng/mL (± 52.18, P3) (363.90 nmol/L), and after 21 days of SuK (P2), 112.70 ng/mL (± 58.46, P2) (310.90 nmol/L). It has been suggested that chronic gluconeogenesis can induce a “fight or flight” emergency survival process, resulting in chronic elevated cortisol secretion [73]. Interestingly, although there were no significant changes in cortisol in the KetoSAge trial, the overall healthy low levels of cortisol should be noted, which indicated that ketosis via carbohydrate restriction with subsequent reliance on gluconeogenesis for a long period of time did not appear to result in hypercortisolaemia. Furthermore, as the KetoSAge participants were, on average, in long-term ketosis for 3.9 years, their plasma glucose source was predominantly from gluconeogenesis, despite having perfectly healthy cortisol levels. Together, these data strongly negate claims that long-term ketosis gluconeogenesis is a fight or flight emergency survival process.

OCN has been shown to modulate insulin sensitivity and secretion via stimulating β-cell serotonin synthesis and secretion to modulate pancreatic islet α cells [8,39], however, this modulation of serotonin may be intracellular, and its secretory actions act in a paracrine manner within the pancreas and, thus, are undetectable in the blood stream. We did not see significant changes in plasma serotonin in the KetoSAge trial.

### 4.6. GLP-1

The current “blockbuster” drugs on the market for diabetes and weight loss are classed as GLP-1 agonists. Our participants in long-term NK showed significantly higher levels of GLP-1, whilst suppressing ketosis significantly decreased GLP-1. Lifestyle practices that stimulate chronic excess insulin demand and secretion result in lower GLP-1 levels and, therefore, less GLP-1 receptor (GLP-1R) activation, which is associated with increased insulin resistance, obesity, and T2DM [74]. Interestingly, in a competing risk regression analysis of 462 incident first cancer cases with 2,417 controls, it was found that a higher fasting GLP-1 level was significantly associated with a lower risk of incident first cancer (sub-hazard ratio 0.90; 95% CI 0.82–0.99; *p* = 0.022) [75].

GLP-1 is excreted primarily by ileo-colonic (L) enteroendocrine cells in the distal small bowel and colon [76]. Interestingly, GLP-1 stimulates proinsulin gene expression, resulting in activating the replenishment of insulin stores [77]. GLP-1 activates GLP-1R on pancreatic islet α cells, inhibiting glucagon secretion, resulting in a lowering of plasma glucose independent of insulin [78,79]. We were unable to measure glucagon in our participants’ samples due to the low sensitivity of the assays trialled, and potentially as a result of glucagon’s rapid degradation ex vivo [80]. However, fasting glucose and insulin were both significantly lower in both NK phases, along with a significantly higher concentration of GLP-1, whilst fasting glucose was significantly higher during the P2 suppression of ketosis with significantly lower GLP-1 levels and higher fasting insulin. This suggests that the lower fasting glucose levels during both NK phases were likely due to the GLP-1 lowering of glucagon, thus resulting in a lower fasting glucose, independent of insulin, and, subsequently, the NK phases having lower fasting insulin values. GLP-1 is an incretin, however, and it enhances insulin secretion in a glucose-dependent manor [81]. Participants’ low HOMA-IR values in both NK phases were half those of when suppressing ketosis (P2), meaning they had a significantly greater insulin sensitivity when in ketosis, which would mean that less basal insulin was required to “do the job” of regulating plasma glucose, if one were to ascribe this as the primary role of insulin. However, in the long-term NK state, basal insulin’s primary role is not likely to be the regulation of plasma glucose and may instead be the regulation of fat oxidation and ketogenesis, as well as modulating hormones and growth factors [2,7], whilst GLP-1 may regulate basal glucose levels via glucagon.

OCN, more specifically, the uncarboxylated form unOCN, stimulates GLP-1 synthesis via the promiscuous GPRC6A receptor [82]. However, the KetoSAge participants had lower OCN (all forms) in both NK phases with higher GLP-1, indicating that the elevated GLP-1 was not likely to be due to endogenous OCN action. As the incretin effect of GLP-1 is glucose dependent, therefore, elevated basal GLP-1 in long-standing euketonaemia is likely acting on its none-incretin actions, such as the slowing of gastric emptying [83]. This may aid in increasing nutrient uptake during food scarcity periods, or when only consuming one meal a day, to facilitate maximal nutrient absorption which would confer a survival benefit. The slowing of gastric emptying may assist in extending the time provided for intestinal processing and maximising the absorption of nutrients from the acidic chyme. This may explain the evolutionary purpose of elevated basal GLP-1 levels in humans who likely spent a great amount of time in ketosis and, thus, not exposed to dietary farinaceous nor sucrose-rich foods that would trigger bolus GLP-1 secretion.

Euketonaemia is achievable without fasting through carbohydrate restriction, which results in a fasting-mimicking state. Palmitic-acid-9-hydroxy-stearic-acid (9-PAHSA), a branched fatty acid ester of hydroxy fatty acids (FAHFAs), stimulates GLP-1 secretion. 9-PAHSA is endogenously synthesised and regulated by a fasting state (euketonaemia/NK) and dietary saturated fatty acid consumption [84]. Insulin-resistant people have lower levels of 9-PAHSA in their serum and adipose tissue, and serum 9-PAHSA levels are significantly correlated with insulin sensitivity shown in humans assessed via euglycaemic clamp [84]. FAHFAs are found in the fat of ruminant meat, milk, eggs, fish [85,86], and breast milk, although they are lower in the breast milk of obese lactating mothers [87]. Interestingly, omnivores have significantly higher serum levels of FAHFA than vegetarians/vegans, and in a 1-week over-feeding study in 15 lean males with a BMI < 27, the consumption of saturated fatty acids from whipping cream was shown to increase serum FAFHA [88,89]. All of these foods are common in many ketosis-supportive lifestyles.

The stimulation of basal GLP-1 secretion has been mainly studied in people in suppressed ketosis (hypoketonaemia) states, who are in the lean and overweight categories, or in pathology. It is arguable that stimulators of basal GLP-1 secretion should be studied in healthy long-term sustained NK (euketonaemia) individuals, who may better reflect the metabolic state that humans likely evolved in, where they would have had less frequent meals, with only seasonally available low farinaceous carbohydrate availability, and, thus, would have been naturally in ketosis for a greater number of hours in their 24 h day and overall year [2,7,39,90,91].

Trials administering oral glucose to hypoketonaemic participants have shown dietary glucose-causing bolus-associated GLP-1 secretion [79]. During the NK phases of the KetoSAge trial, the participants did not consume enough carbohydrates to cause the suppression of ketosis, whilst they consumed 267 g of carbohydrate spread over three meals a day during phase 2 to suppress ketosis. As can be seen, basal GLP-1 was significantly higher in the two phases with the least dietary glucose exposure, whilst after the participants had followed the SUK dietary guidelines to habitually consume the equivalent of three OGTTs worth of glucose for 21 days straight, which would be equivalent to 63 OGTTs, the KetoSAge participants’ mean basal fasting GLP-1 levels were significantly lower, with higher fasting glucose and insulin. This might be explained by increased GLP-1 clearance in a hypoketonaemia state, via increased DDP-4 enzymatic breakdown or increased renal clearance. Alternatively, euketonaemia may decrease DDP-4 activity, thus reducing GLP-1 clearance [83,92]. This warrants further investigation.

Given that the “blockbuster” drugs for the current treatment for T2DM, and now used for the treatment of obesity to induce weight loss, are GLP-1R agonists [93], it would be beneficial to understand the physiological role of basal GLP-1 in healthy humans in a metabolic state reflective of how we evolved, namely, euketonaemia MP1. The fasting-mimicking-state euketonaemia effectuates weight normalisation, shown to bring back weight homeostasis, meaning that the overweight will lose weight and the underweight will return to a healthier weight, whilst resolving many metabolic and neurological health conditions in the process [94,95,96,97,98,99].

## 5. Strengths and Limitations

Our study is a novel investigation of the biomarkers in chronic diseases of ageing in a healthy pre-menopausal female population living in long-term ketosis. OCN and GLP-1 have not been measured in this cohort before. Further work assessing these biomarkers in larger cohorts across different ages and in pathologies should be considered. Due to the low sensitivity of several trialled glucagon assays, we were unable to measure glucagon in this cohort of participant samples. Further details on strengths and limitations have been previously discussed in detail in our earlier publication [7].

## 6. Conclusions

Long-term ketosis does not result in chronic elevated cortisol levels, indicating that ketosis is not a chronic stress-inducing nor fight or flight state. Long-term NK, euketonaemia, results in elevated GLP-1 levels that would presumably stimulate GLP-1R, which is associated with maintaining a healthier weight or weight loss and satiety, a method employed by T2DM and weight loss GLP-1R agonists. Long-term sustained euketonaemia results in lower levels of leptin, which is associated with increased satiety sensitivity and healthier metabolic profiles. Whilst OCN is considered to be necessary for insulin secretion, given that we understand that a low level of insulin that does not suppress ketogenesis is a healthy level of insulin, MP1, euketonaemia, presents with lower plasma OCN levels. This likely indicates that the OCN remains in the bone, and would confer greater bone health. This is understood to be corroborated in those with low HOMA-IR values also having a lower risk of fragility fractures, indicating insulin sensitivity and, thus, not being hyperinsulinaemic. Furthermore, the increased plasma OCN seen after suppressing ketosis may be from osteoclast bone resorption, which may, over time, result in the hyper-mineralisation of the bones (increased BMD seen in T2DM and obesity), but also be coupled with an increased brittleness, resulting in increased fragility fractures rate, as seen in obesity and T2DM individuals. Lower OCN levels are found in the healthy euketonaemic state, and are accompanied with lower fasting glucose, insulin, insulin sensitivity HOMA-IR, and leptin and higher GLP- 1. Sustained NK showed no adverse health effects and may mitigate hyperinsulinemia. All biomarkers returned to basal P1 levels after removing the intervention for SuK, indicating that metabolic flexibility is maintained with long-term euketonaemia.

## Figures and Tables

**Table 2:** BMI, fat mass, fasted insulin, glucose, BHB, HOMA-IR, total, carboxylated and uncarboxylated osteocalcin, leptin, cortisol, serotonin, and GLP-1 across all phases in KetoSAge participants. Measurements were taken following each of the study phases: baseline nutritional ketosis (NK), P1; intervention suppress ketosis (SuK), P2; and removal of SuK returning to NK, P3. Measurements were taken at 8 a.m. after a 12 h overnight fast; (n = 10). Values are presented as mean ± SD.

	P1	P2	P3	ANOVA *p* Value	P1 vs. P2	P2 vs. P3	P1 vs. P3
BMI (kg/m^2^)	20.52 (±1.39)	21.54 (±1.30)	20.82 (±1.46)	<0.0001	<0.0001	0.0025	0.0197
Fat mass (kg)	14.21 (±2.55)	15.88 (±2.23)	14.78 (±2.20)	<0.0001	0.0008	0.0057	0.1016
Insulin (µIU/mL)	4.95 (±1.24)	9.06 (±2.14)	5.62 (±1.83)	<0.0001	0.0006	0.0027	0.3995
Glucose (mmol/L)	4.36 (±0.53)	5.12 (±0.59)	4.41 (±0.30)	0.0015	0.0088	0.0177	0.9469
BHB (mmol/L)	2.43 (±1.28)	0.18 (±0.13)	2.31 (±0.71)	0.0001	0.0012	<0.0001	0.9854
HOMA-IR	0.97 (±0.32)	2.07 (±0.61)	1.11 (±0.41)	<0.0001	0.0008	0.0013	0.4950
tOCN (ng/mL)	33.84 (±13.66)	55.31 (±29.71)	34.02 (±12.05)	0.0049	0.0138	0.0253	0.9978
cOCN (ng/mL)	31.54 (±12.59)	50.78 (±26.22)	32.02 (±11.13)	0.0040	0.0120	0.0246	0.9840
unOCN (ng/mL)	2.29 (±1.25)	4.54 (±3.79)	2.00 (±1.16)	0.0004	0.0417	0.0010	0.7907
tOCN % (% relative to P1)	100.00	162.36 (±46.53)	109.70 (±40.36)	0.0005	0.0065	0.0094	>0.9999
cOCN % (% relative to P1)	100.00	161.56 (±46.73)	111.52 (±42.90)	0.0007	0.0062	0.0080	0.6836
unOCN % (% relative to P1)	100.00	176.67 (±66.66)	87.11 (±21.43)	0.0028	0.0135	0.0076	0.2476
Leptin (ng/mL)	4.50 (±3.67)	15.08 (±8.00)	4.57 (±3.48)	<0.0001	0.0010	0.0052	>0.9999
Cortisol (ng/mL)	126.20 (±52.67)	112.70 (±58.46)	131.90 (±52.18)	0.4362	>0.9999	0.5391	>0.9999
Serotonin (ng/mL)	21.05 (±22.83)	18.38 (±16.03)	21.77 (±16.80)	0.6013	0.7907	>0.9999	>0.9999
GLP-1 (pg/mL)	1383.18 (±911.36)	576.72 (±452.43)	1471.85 (±1066.75)	0.0075	0.0209	0.0219	>0.9999

Beta-hydroxybutyrate (BHB); body mass index (BMI); carboxylated osteocalcin (cOCN); glucagon like peptide 1 (GLP-1); homeostasis model assessment for insulin resistance (HOMA-IR); total osteocalcin (tOCN); and uncarboxylated osteocalcin (unOCN).

## Data Availability

The data presented in this study are available from the corresponding author upon reasonable request.

## Abbreviations

9-PAHSA	palmitic-acid-9-hydroxy-stearic-acid
ACD	all-cause dementia
AD	Alzheimer’s disease
BA	biological ageing
BHB	beta-hydroxybutyrate
BMI	body mass index
CAC	coronary artery calcification
CI	confidence interval
cOCN	carboxylated osteocalcin
CVD	cardiovascular disease
EPC	endothelial progenitor cells
FAHFA	fatty acid ester of hydroxy fatty acids
GGT	gamma-glutamyl transferase
GLP-1	glucagon like peptide-1
GLP-1R	glucagon like peptide-1 receptor
HA	hydroxyapatite
HOMA-IR	homeostatic model assessment for insulin resistance
IGF-1	insulin like growth factor-1
L	ileo-colonic
MASLD	metabolic-dysfunction-associated steatosis liver disease
MCP-1	monocyte chemotactic protein-1
MetS	metabolic syndrome
Mt	mitochondrial
MP	metabolic phenotype
NK	nutritional ketosis
OGTT	oral glucose tolerance test
OR	odds ratio
OXPHOS	oxidative phosphorylation
P1	Phase 1
P2	Phase 2
P3	Phase 3
RCT	randomised control trial
SA	sub-clinical atherosclerosis
SFA	saturated fatty acid
SUK	standard U.K. diet
SuK	suppression of ketosis
TOFI	thin on the outside fat on the inside
T2DM	type 2 diabetes mellitus
T3	triiodothyronine
tOCN	total osteocalcin
unOCN	uncarboxylated osteocalcin
